# Targeting Herpes Simplex Virus-1 gD by a DNA Aptamer Can Be an Effective New Strategy to Curb Viral Infection

**DOI:** 10.1016/j.omtn.2017.10.009

**Published:** 2017-10-17

**Authors:** Tejabhiram Yadavalli, Alex Agelidis, Dinesh Jaishankar, Kyle Mangano, Neel Thakkar, Kumar Penmetcha, Deepak Shukla

**Affiliations:** 1 Department of Ophthalmology and Visual Sciences, University of Illinois at Chicago, Chicago, IL 60612, USA; 2 Department of Microbiology and Immunology, University of Illinois at Chicago, Chicago, IL 60612, USA; 3 Department of Bioengineering, University of Illinois at Chicago, Chicago, IL 60607, USA; 4 Medicinal Chemistry and Pharmacognosy, University of Illinois at Chicago, Chicago, IL 60612, USA; 5 Biomedical Research Institute, National Institute of Advanced Industrial Science and Technology (AIST), Tsukuba Science City, Ibaraki 305-8566, Japan

**Keywords:** DNA aptamer, topical treatment, herpes simplex virus 1, prophylaxis, therapy

## Abstract

Herpes simplex virus type 1 (HSV-1) is an important factor for vision loss in developed countries. A challenging aspect of the ocular infection by HSV-1 is that common treatments, such as acyclovir, fail to provide effective topical remedies. Furthermore, it is not very clear whether the viral glycoproteins, required for HSV-1 entry into the host, can be targeted for an effective therapy against ocular herpes *in vivo*. Here, we demonstrate that HSV-1 envelope glycoprotein gD, which is essential for viral entry and spread, can be specifically targeted by topical applications of a small DNA aptamer to effectively control ocular infection by the virus. Our 45-nt-long DNA aptamer showed high affinity for HSV-1 gD (binding affinity constant [K_d_] = 50 nM), which is strong enough to disrupt the binding of gD to its cognate host receptors. Our studies showed significant restriction of viral entry and replication in both *in vitro* and *ex vivo* studies. *In vivo* experiments in mice also resulted in loss of ocular infection under prophylactic treatment and statistically significant lower infection under therapeutic modality compared to random DNA controls. Thus, our studies validate the possibility that targeting HSV-1 entry glycoproteins, such as gD, can locally reduce the spread of infection and define a novel DNA aptamer-based approach to control HSV-1 infection of the eye.

## Graphical Abstract

**Figure:**
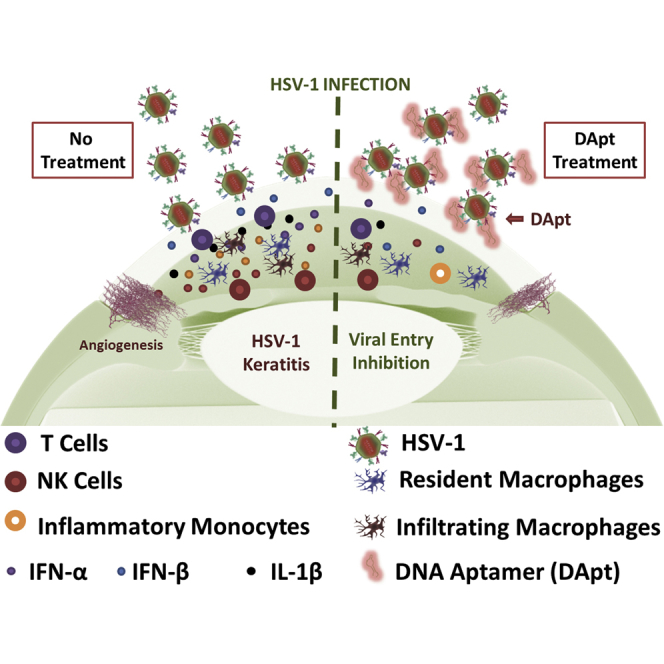

## Introduction

Herpes simplex virus type-1 (HSV-1), belonging to the family *Herpesviridae*, causes herpes labialis and ocular keratitis, which is one of the main causes of infectious blindness in the US.^1^ HSV-1 has a global sero-prevalence in the range of 60%–90%, with recurrent infections causing corneal scarring, neovascularization, and stromal keratitis.^2^, ^3^ HSV-1 might also lead to other more serious diseases, such as retinitis, encephalitis, and sporadic cases of systemic morbidities, especially among immunocompromised patients.^4^ HSV-1 consists of a double-stranded DNA genome enclosed within an icosahedral capsid that is surrounded by a double layered lipid membrane envelope.^5^ The viral envelope is covered with a dozen different glycoproteins that include four essential glycoproteins, namely, gB, gD, and gH/gL, which facilitate virus host membrane fusion required for virus entry and cell-to-cell spread.^6^ The glycoprotein gD is essential for interaction with host receptors, leading to viral entry and/or spread. It binds to the cell surface receptors nectin-1, HVEM, and 3-*O* sulfated heparan sulfate with similar affinity, and the binding affinity constant (K_d_) values appear to be in the low micromolar to higher nanomolar range.^7^ Because gD is essential for viral infectivity, is abundantly expressed on the HSV-1 envelope and the plasma membrane of infected cells, and does not share homologies to any known host cell proteins, it can be an ideal candidate for antiviral drug targeting.

Nucleic acid aptamers are single-stranded oligonucleotides that provide unprecedented binding specificity and equally strong affinity to a variety of targets, including inorganic molecules, cellular proteins, and viral glycoproteins.^8^, ^9^, ^10^, ^11^ Their sizes range from a few nucleotides to a few hundred nucleotides. Among many types of applications, medical or non-medical, aptamers can also be used for target-based topical therapies for HSV-1 infection. Such alternative treatments are crucial because conventional anti-HSV therapies, such as acyclovir, and other nucleoside analogs cause serious side effects and are prone to the development of viral resistance.^12^ Similar issues exist with the treatment of HSV-1 infection of the eye, which can linger for months in many cases, and topical treatments fail to control the ocular disease manifestations. So far there have been only two studies reporting the use of aptamers for neutralizing HSV infectivity. Our collaborators reported the use of an RNA aptamer in cell cultures that targeted HSV-1 gD^13^ and Moore et al.^14^ reported the use of another RNA aptamer that targeted the gD protein of HSV-2. The original RNA aptamer developed by our collaborators was 113 nt long and highly specific for HSV-1 gD because it did not block infection of HSV-2 virions.^13^

Given their ability to acquire a high degree of structural divergence and ease of selection against any target of interest, RNA aptamers offer many advantages over newer technologies that currently exist.^8^, ^9^, ^10^, ^11^ However, two major disadvantages include lack of stability and higher manufacturing costs, which can hinder commercialization efforts. DNA aptamers, on the other hand, can be more stable, easier to manufacture, and more cost effective. Therefore, in our quest to study HSV-1 gD in viral entry and spread *in vivo* and also to develop an effective topical therapy against ocular herpes, we designed and tested a DNA aptamer (DApt) that derives its sequence from the mini-1 RNA aptamer used by our collaborators.^13^ The DNA-based design of this aptamer preserves the functional characteristics of the mini-1 RNA aptamer.^13^ Here, we show the target specificity and antiviral efficacy of DApt using corneal cell cultures, corneal organ cultures, and mice models of ocular infection. To the best of our knowledge, ours is the very first report demonstrating the effect of blocking gD on HSV-1 infectivity in the eye and the use of DNA aptamers as viral entry blocking agents against HSV-1. It is also the most thorough study using any kind of aptamer against herpesviruses.

## Results

### Identification and Structural Characterization of DApt that Binds to HSV-1 gD

**Figure 1:**
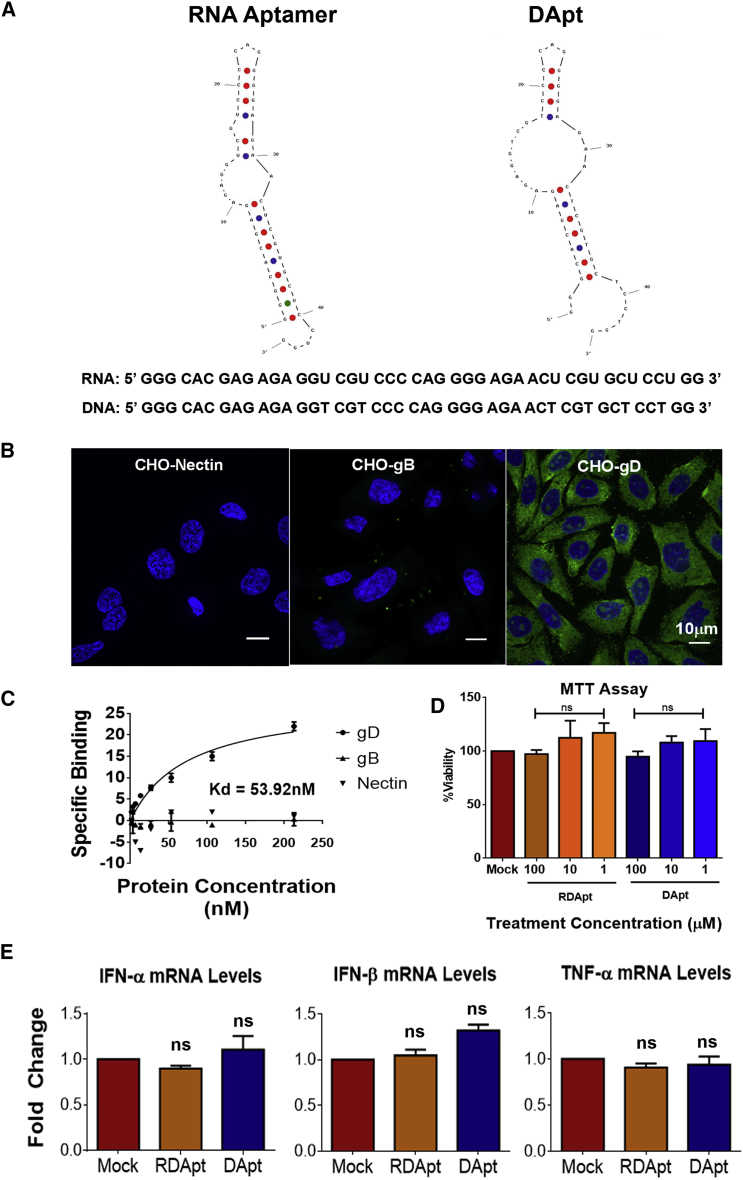
Physio-chemical and Biological Properties of the Aptamer (A) The functional sequence of the RNA aptamer with preserved gD protein-binding affinity is shown, along with the DNA aptamer that was designed from the same. Both aptamers have a similar structure, as evaluated by the OligoAnalyzer tool available from IDT. (B) Representative confocal images of CHO cells expressing either Nectin (left), HSV-1 gB (middle), or HSV-1 gD (right) viral glycoprotein and incubated with FAM-modified DApt (GFP). CHO cells were transfected using lipofectamine protocol with host protein Nectin-1, viral glycoprotein gB, or viral glycoprotein gD for 24 hr. The cells were then fixed and permeabilized before they were incubated with FAM-tagged DApt (GFP) for 30 min to initiate attachment between DApt and target proteins. Scale bar is the same for all images. (C) Binding affinity was determined using a modified SYBR green assay. Varying concentrations of protein (Nectin, gB, or gD) were incubated with DApt in a 96-well plate for a period of 30 min before SYBR green was added to each well. Unbound DApt would sequester SYBR green to produce fluorescence, which was recorded using a fluorescence spectrometer. Change in fluorescence was used as an estimate to calculate specific binding of DApt to the mentioned proteins. The specific K_d_ shown on the graph was calculated using standardized non-linear regression analysis using GraphPad Prism software for the interaction between DApt and gD protein. (D) Aptamer toxicity was assessed using an MTT assay on HCEs that were incubated with indicated concentrations of PBS, DApt, and RDApt for a period of 24 hr. Data are represented as mean ± SD. The values have been normalized to mock-treated samples. (E) Pro-inflammatory cytokine analysis via qRT-PCR on HCEs incubated with 2 μM of indicated treatments for 24 hr. Data are represented as mean ± SD. Data points were normalized to GAPDH. One-way ANOVA with Dunnett’s multiple comparison test, with a single pooled variance: p < 0.0001.

In order to study the significance of inhibiting gD on HSV-1 infectivity *in vivo*, we designed and tested the DNA version of an RNA aptamer that very specifically targets HSV-1 gD.^13^ We reasoned that a DNA aptamer, which preserves many features of the RNA aptamer, may provide a more viable therapeutic option against HSV-1 infection. The sequence of DApt was derived after mini-1 RNA aptamer, which contains the gD-binding region of the original RNA aptamer sequence.^13^ The sequence for the short RNA aptamer that preserves most of the antiviral activity of the parent RNA aptamer and its DNA replica are shown in Figure 1A. The gD-binding ability of the mini-1 RNA aptamer was already reported to be similar to that of the parent RNA aptamer.^13^ The secondary structures of the mini-1 RNA aptamer and DApt were predicted using the IDT OligoAnalyzer tool. The two sequences appear to preserve the loop structures that are important for interaction with gD.^13^ The distance between the loops provides sufficient flexibility for the aptamers to conform to the receptor-binding region of the surface exposed 3-dimensional structure of gD.^15^ Based on similar studies, the structural flexibility of DApt may be slightly lower than its RNA homolog, which, as shown below, did not affect its antiviral properties against HSV-1.^16^, ^17^ The entropy values were predicted as −443.01 and −294 cal.K^−1^ mol^−1^ for RNA and DApt, respectively. The latter suggests a higher thermostability and shelf life for DApt.

### DApt Binds Specifically to HSV-1 gD

DApt-binding affinity toward gD protein was evaluated along with two control proteins. The controls used included a cell surface gD receptor protein, nectin-1, and HSV-1 envelope glycoprotein, gB. To verify the binding specificity, we conducted an immunofluorescence study using FAM-tagged DApt (Integrated DNA Technologies, USA) to assess its binding specificity.^18^ Chinese hamster ovary (CHO) cells transfected with either Nectin-1 or HSV-1 glycoproteins gD or gB were permeabilized and incubated with FAM-DApt for a period of 30 min before immunofluorescence imaging. Only the cells expressing gD viral protein had the presence of FAM-aptamer compared to cells expressing empty vector (not shown), Nectin-1, or gB (Figure 1B). This assay has a big advantage in that it examines binding of a ligand (aptamer) against a cell-surface-expressed full-length protein, which is present in its native form. All other gD-binding assays use a purified but truncated form of gD, which may not be folded exactly as its membrane-bound native form. The binding specificity of the aptamer to gD protein was further quantified via a modified SYBR Green (SG) assay.^19^ Because the intensity of SG varied based on the availability of free aptamers (not bound to protein) in the solution, a nonlinear regression analysis of the determined values was plotted to give a specific K_d_ of 53.92 nM (Figure 1C). Taken together, these results indicate that DApt binds to glycoprotein gD, albeit with a lower affinity than its parent mini-1 RNA aptamer (4 nM).

### DApt Is Non-toxic and Does Not Induce Host Response

To rule out cytotoxicity of DApt, an MTT (3-(4,5-dimethylthiazol-2-yl)-2,5-diphenyltetrazolium bromide) assay was performed. Human corneal epithelial cells (HCEs) were treated with the indicated concentrations of DApt and a random DNA aptamer sequence (RDApt) was used as a control. The results (Figure 1D) showed no difference in cell viability at concentrations as high as 100 μM. Furthermore, RNA aptamers are known to generate an elevated cytokine response in host cells, which in turn would inhibit viral replication.^16^, ^17^ Hence, to understand the role of our aptamer in influencing host cytokine response, HCEs were incubated with DApt and RDApt aptamers in the absence of an infection. RNA collected from the samples was reverse transcribed to DNA and evaluated for any change in regulatory cytokines, such as interferon α (IFN-α), IFN-β, and tumor necrosis factor alpha (TNF-α). No significant change in cytokine levels was observed in cells exposed to either DApt or RDApt when compared to the mock-treated cells (Figure 1E). These results validate that any antiviral activity shown by DApt would in fact be due to viral protein neutralization and not due to changes in cytokine activity.

### DApt Inhibits HSV-1 Entry

**Figure 2:**
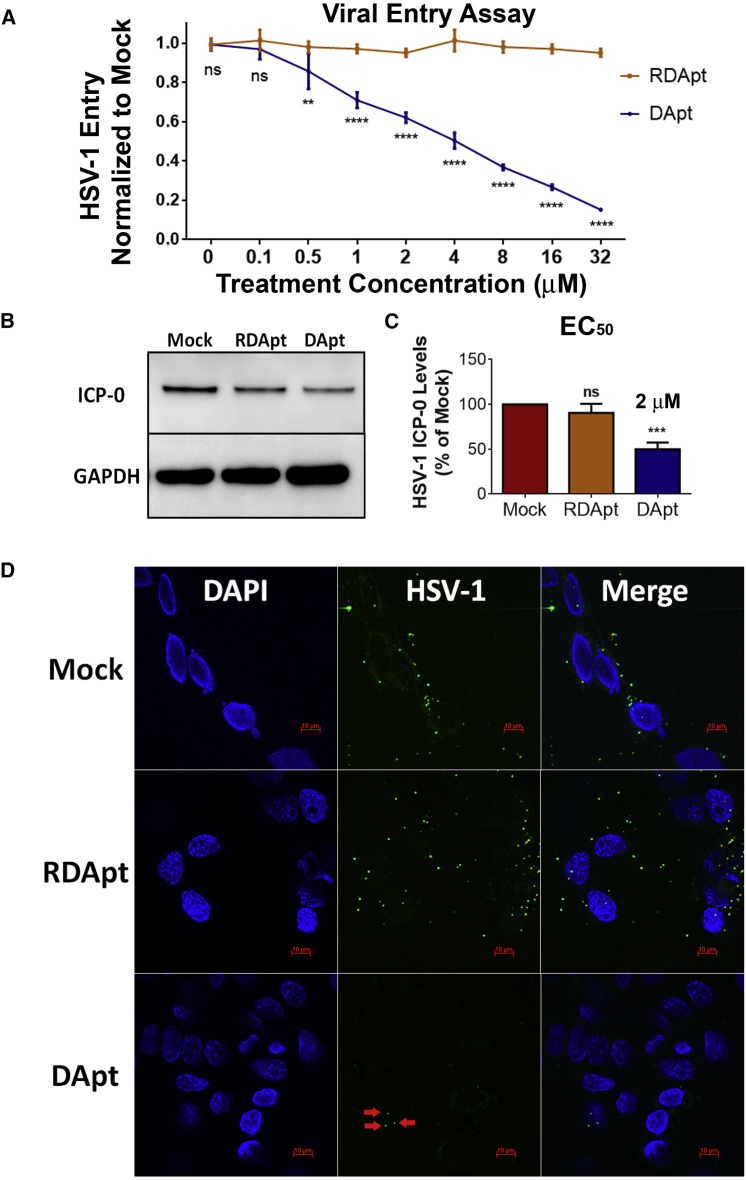
DApt Restricts HSV-1 Entry (A) HSV-1 viral entry into HCEs was assessed using a β-galactosidase-expressing reporter virus. MOI 10 HSV-1 gL86 was neutralized with the DApt/RDApt at the indicated concentrations for 30 min before infecting the cells. Asterisks indicate a significant difference by two-way ANOVA with Sidak’s multiple comparison test: **p < 0.01 and ****p < 0.0001. (B and C) Representative immunoblots (B) and quantification (C) of HSV-1 ICP-0 (early) protein levels at 6 hpi in HCEs infected with MOI 10 HSV-1(KOS). The virus was neutralized with 2 μM (EC_50_) mock/DApt/RDApt for 30 min prior to infection. Asterisks indicate significant difference by one-way ANOVA with Dunnett’s multiple comparison test, with a single pooled variance: ***p < 0.0003. (D) Representative confocal images of HCEs showing the presence of internalized GFP virus in HCEs. HSV-1(17-GFP) at MOI 10 were incubated with either 2 μM mock (PBS)/RDApt/DApt for 30 min at room temperature prior to infecting the cells at 4°C for 2 hr. Viral entry was initiated by placing the cells at 37°C for 30 min before cells were fixed and imaged. (A and C) Data are represented as means ± SD. Scale bar for all images, 10 μm.

Because our aptamer binds gD with high affinity, we predicted that it has the ability to neutralize infectious virions by disrupting interactions with the host receptor. Experiments were conducted by incubating known concentrations of DApt with a β-galactosidase-expressing HSV-1 reporter virus (KOS gL86). DApt restricted viral entry by approximately 50% and 80% at concentrations of 2 μM and 32 μM, respectively, when infected at an MOI of 10 (Figure 2A). Furthermore, immunoblotting of the HSV-1 (17-GFP) infected cell lysate for ICP0 (one of the early viral gene products made immediately upon HSV-1 entry) showed significantly lower levels in cells treated with DApt compared to RDApt (Figures 2B and 2C). Finally, immunofluorescence imaging showed a significant reduction in entry of GFP-tagged HSV-1 (17-GFP) into HCEs treated with DApt (Figure 2D). Collectively, these results indicate that DApt inhibits HSV-1 entry.

### DApt Reduces Overall HSV-1 Infection

**Figure 3:**
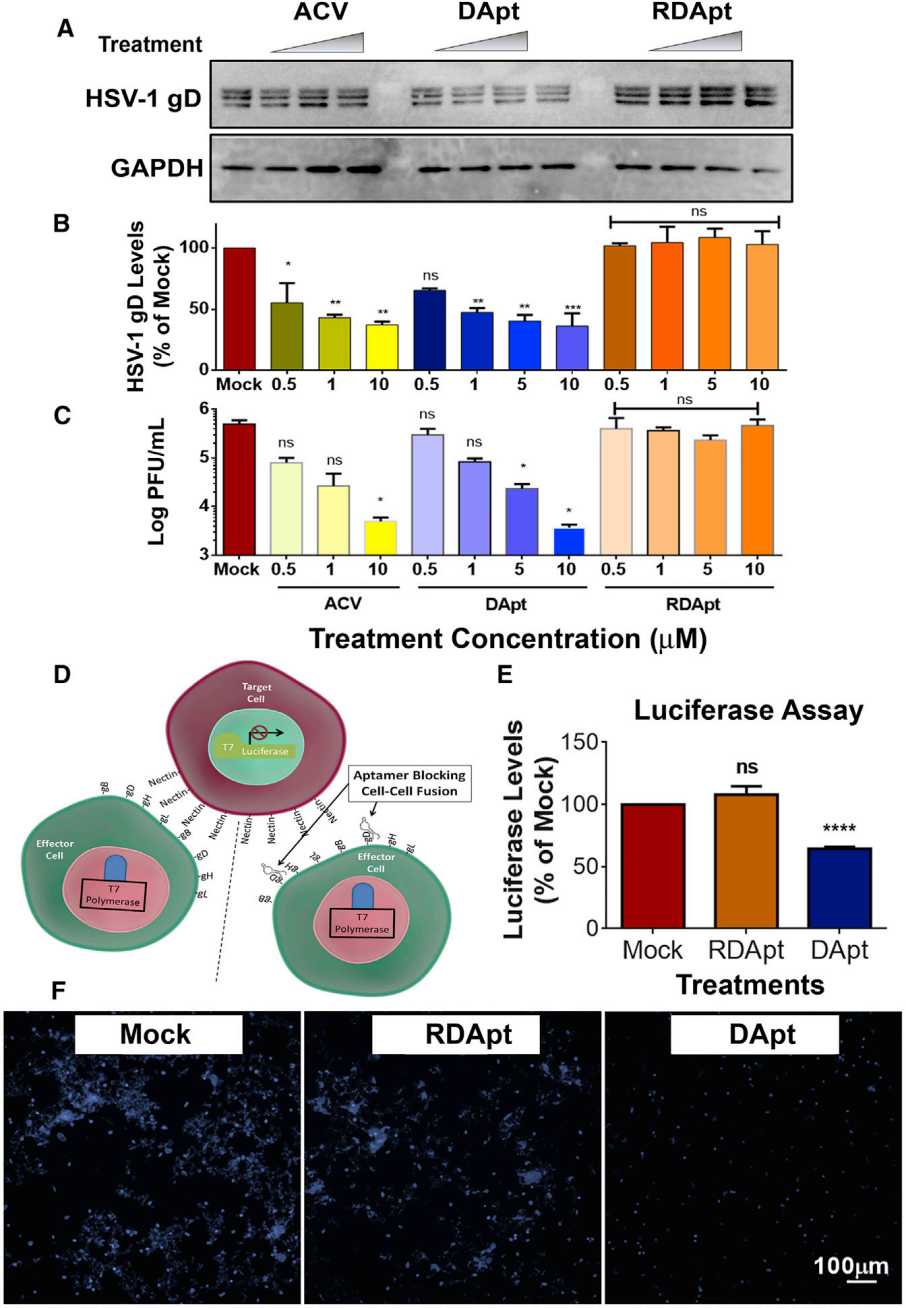
DApt Reduces Viral Replication and Minimizes Cell-to-Cell Spread (A–C) Representative immunoblots (A), quantification of HSV-1 gD protein levels (B), and supernatant plaque assays (C) at 24 hpi in HCEs infected with HSV-1(KOS) at MOI 1. The virus was neutralized with indicated concentrations of mock(PBS)/ACV/DApt/RDApt for a period of 30 min prior to infection. Asterisks indicate significant difference by one-way ANOVA with Dunnett’s multiple comparison test, with a single pooled variance: *p < 0.05, **p < 0.01, and ***p < 0.001. (D) Schematic of cell-to-cell fusion assay. CHO cells were categorized into two populations: effector (green) and target (red) cells. Effector cells express HSV-1 glycoproteins (gD, gB, gH, and gL) and T7 polymerase, whereas the target cells express nectin-1 (a gD receptor) and the luciferase gene under T7 promoter. Luciferase activity was detected when the cells fuse. (E) Luciferase values representing fusion of CHO cells. Effector CHO cells were treated with either 2 μM mock (PBS)/DApt/RDApt for 30 min before they were mixed with target CHO cells. Asterisks indicate significant difference by one-way ANOVA with Dunnett’s multiple comparison test, with a single pooled variance: ****p < 0.0001. (F) Representative fluorescence microscopy images of fused CHO cells showing the presence of syncytial cluster formation (in blue). Effector CHO cells were pre-treated with 2 μM mock (PBS)/DApt/RDApt for 30 min before they were added to the target CHO cells. Images of the syncytial cluster were taken by dyeing the cells with NucBlue live cell nucleus stain. Scale bar is similar for all images. (B, C, and E) Data are represented as means ± SD.

Because DApt blocks viral entry, it should reduce the number of virions entering into cells, which, in turn, should result in loss of viral infectivity. To test this, HCEs were infected with HSV-1(KOS) at MOI of 1 after they were neutralized with DApt/RDApt/acyclovir at indicated concentrations. At 24 hr post infection (hpi), cell lysates and cell supernatant (containing released virus) were collected for immunoblotting and viral titer analysis, respectively. We observed a decrease in viral protein (gD) by approximately 50% and 70% at concentrations of 5 μM and 10 μM, respectively (Figures 3A and 3B). We also saw significantly lower released viral titers in these samples (Figure 3C). Similar experiments at varied MOIs and lower aptamer concentrations were conducted with similar results (Figures S1A, S1B, S2A, and S2B). To further understand if DApt would have neutralizing properties against acyclovir-resistant strains, similar experiments were conducted using the HSV-1 TK-12 strain, which is an acyclovir-resistant strain because it lacks viral thymidine kinase, the molecular target of acyclovir. We found that there was no significant difference in the neutralizing ability of the aptamer between acyclovir-sensitive or -resistant strain (Figures S1C and S1D). In all cases, the aptamer showed a similar virus neutralizing potential, clearly suggesting that an aptamer-based therapy will work equally well against acyclovir-resistant strains.

### DApt Restricts Cell-to-Cell Fusion

It is well known that after infection, HSV-1 spreads from one cell to another by enabling membrane fusion to cause multinucleated syncytia formation, which requires gD and its receptors.^20^ Because DApt blocks gD with a much higher affinity than gD’s affinity for any of its receptors,^7^ we hypothesized that DApt should have the ability to disrupt HSV-1-mediated membrane fusion. The effect on membrane fusion was studied both visually and through a luciferase-based reporter assay described previously^21^, ^22^ at the half maximal effective concentration (EC_50_) of DApt. Target cells expressing the entry receptor nectin-1 and luciferase gene were mixed with the effector cells expressing viral glycoproteins and T7 RNA polymerase (Figure 3D). Fusion was monitored as a function of luciferase activity on its substrate (Figure 3E) and in parallel visualized by staining the cellular nuclei with DAPI stain (Figure 3F). As hypothesized, fusion was restricted to a significant extent (35%) by DApt compared to RDApt and mock-treated cells. This indicates that DApt not only restricts extracellular entry of the virus, but it can also block intracellular spread by restricting fusion pore formation between neighboring cells, which could be a mechanism behind the therapeutic effects of DApt against existing HSV-1 infections.

### DApt Restricts Spread and Infection in *Ex Vivo* Corneal Model

**Figure 4:**
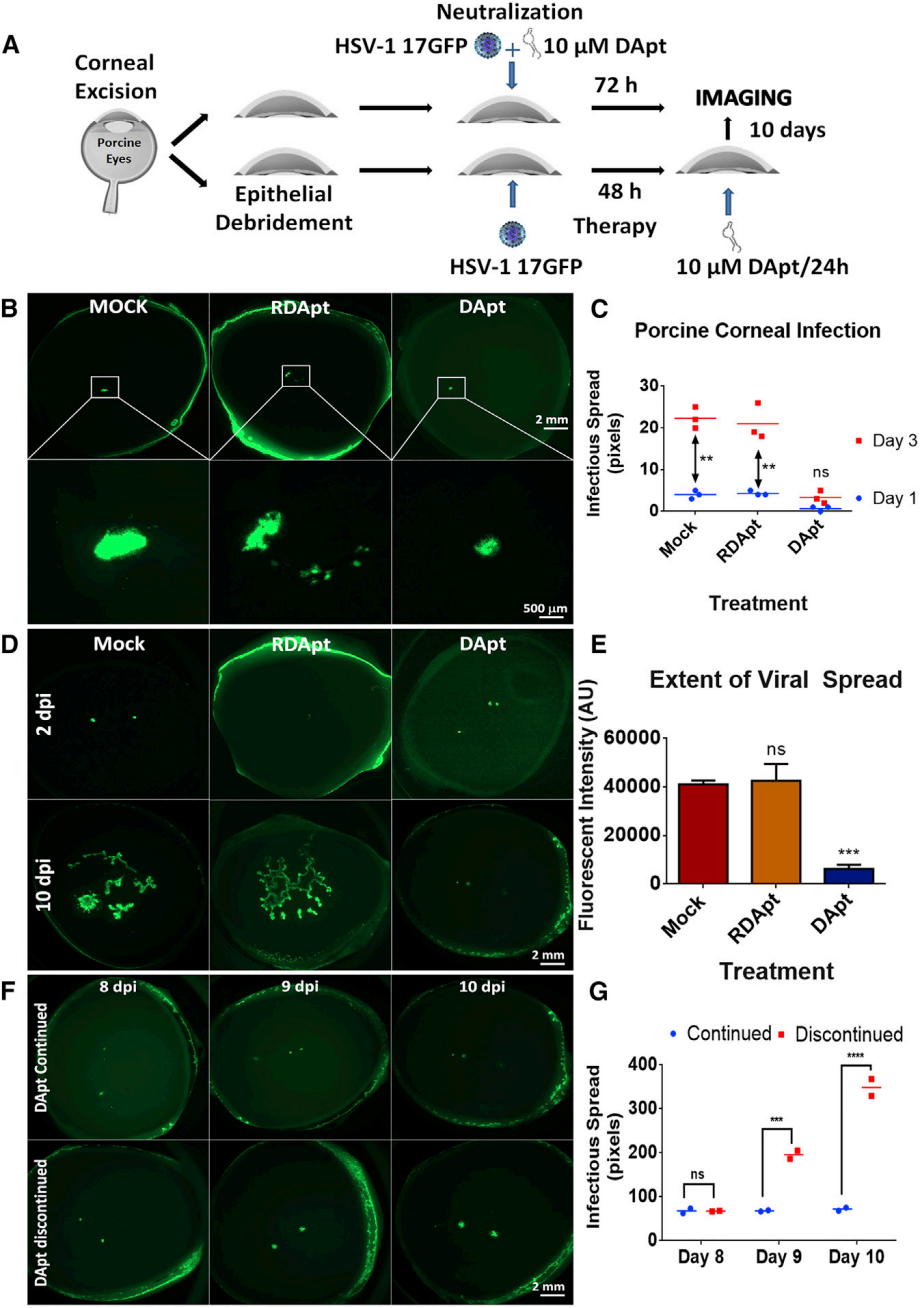
DApt Reduces HSV-1 Infection in the *Ex Vivo* Models (A) A schematic of the *ex vivo* model. Porcine corneas were excised and poked (at the center of the cornea) with a 30G needle to cause epithelial debridement. Following this, the porcine corneas were infected and treated by DApt to check for its neutralization capabilities or therapeutic efficacy. (B) Representative porcine corneal images showing the presence of virus (green) at 72 hpi. The figures shown on the top half were imaged at 7.5× magnification, whereas the bottom half are the magnified images of the same corneas at 32×. 1 × 10^6^ PFU HSV-1(17-GFP) was neutralized with 10 μM mock (PBS)/DApt/RDApt for 30 min prior to infecting the porcine corneas. The corneas were washed with PBS, and media was replenished every 24 hr for 3 days. No additional treatments were added during this period. (C) Quantification of viral spread from the poke site. The plot represents difference in areal spread of infection in individual corneas neutralized by indicated treatments over a 3-day period. Asterisks indicate significant difference by repeated-measures two-way ANOVA with Sidak’s multiple comparison test: **p < 0.01. (D) Representative porcine corneal images showing the presence of virus (green) at indicated times. Porcine corneas were infected with 10^6^ PFU HSV-1(17-GFP) for 48 hr to initiate infection. Therapeutic treatment was started at 48 hpi by the addition of 10 μM mock (PBS)/DApt/RDApt. The corneas were washed and treatments were then added every 24 hr for 10 days. (E) Quantification of viral spread from the 2 poke sites. The plot represents a difference in fluorescence intensity (virus spread) between corneas treated by indicated treatments for a period of 10 days. Asterisks indicate significant difference by one-way ANOVA with Dunnett’s multiple comparison test, with a single pooled variance: ***p < 0.001. (F) Representative porcine corneal images showing the presence of virus (green) at indicated times. Within the therapeutic model, at 7 dpi, DApt treatment was continued on one set of porcine corneas, whereas the other set was left untreated for 72 hr in order to evaluate changes in infectious spread. Scale bars shown are the same for all the images. (G) Representative fluorescence intensity values for corneas that continued to receive DApt treatment or for which DApt treatment was discontinued. (E) Data are represented as means ± SD.

Based on the evidence gathered in the previous section, an *ex vivo* porcine corneal model was developed to investigate the extent of viral spread in treated and untreated conditions. We and others have demonstrated that cultured porcine corneas can provide an infection model that mimics many key characteristics of human clinical disease.^23^, ^24^ Neutralization and therapeutic studies were conducted using equal amounts of HSV-1 17-GFP virus to infect porcine corneal epithelium. A schematic representing the process of porcine corneal tissue infection and treatment is depicted in Figure 4A. Neutralization was performed by incubating (pre-heated and cooled) DApt with the virus for a period of 30 min before applying them onto the cornea. The site of epithelial debridement (and infection) was closely monitored for a period of 72 hr. Stereoscopic images taken at 72 hpi were analyzed using ImageJ software to quantify the extent of HSV-1 spread in the presence or absence of DApt neutralization. Based on the notion that non-neutralized virus would constitute infection (radial spread of the virus), extent of viral spread (GFP) was monitored for a period of 72 hr in all three treatment groups. We observed significantly lower infection (radial spread of GFP) in DApt-treated corneas when compared to RDApt- and mock-treated corneas (Figures 4B and 4C).

In a separate experiment, the therapeutic efficacy of DApt was evaluated by starting the treatment at 48 hr post epithelial debridement and infection with a GFP virus (HSV-1 17 GFP). DApt, RDApt, or mock (PBS) treatments were applied as eye drops to the cornea every 24 hr, and the progression of infection was monitored by a Zeiss stereoscope. It was evident that mock-treated and RDApt-treated corneas become rampant with infectious spread (green), whereas a very minute spread is observed in the DApt-treated corneas (Figure 4D). Although viral (green) spread was observed in DApt-treated corneas, it was significantly lower when compared to its counterparts (Figure 4E).

To understand whether the discontinuation of DApt treatment would lead to an increase in viral spread, 7 days post infection (dpi), one set of corneas (previously treated with DApt) was left untreated for 72 hr, whereas the other sets were continued on DApt treatment. As expected, we saw an increase in viral spread in the treatment discontinued cornea compared to the treated ones (Figures 4F and 4G), indicating that DApt was able to continuously restrict viral spread during this time frame.

### DApt Reduces Infectious Spread of HSV-1 *In Vivo* Corneal Models

**Figure 5:**
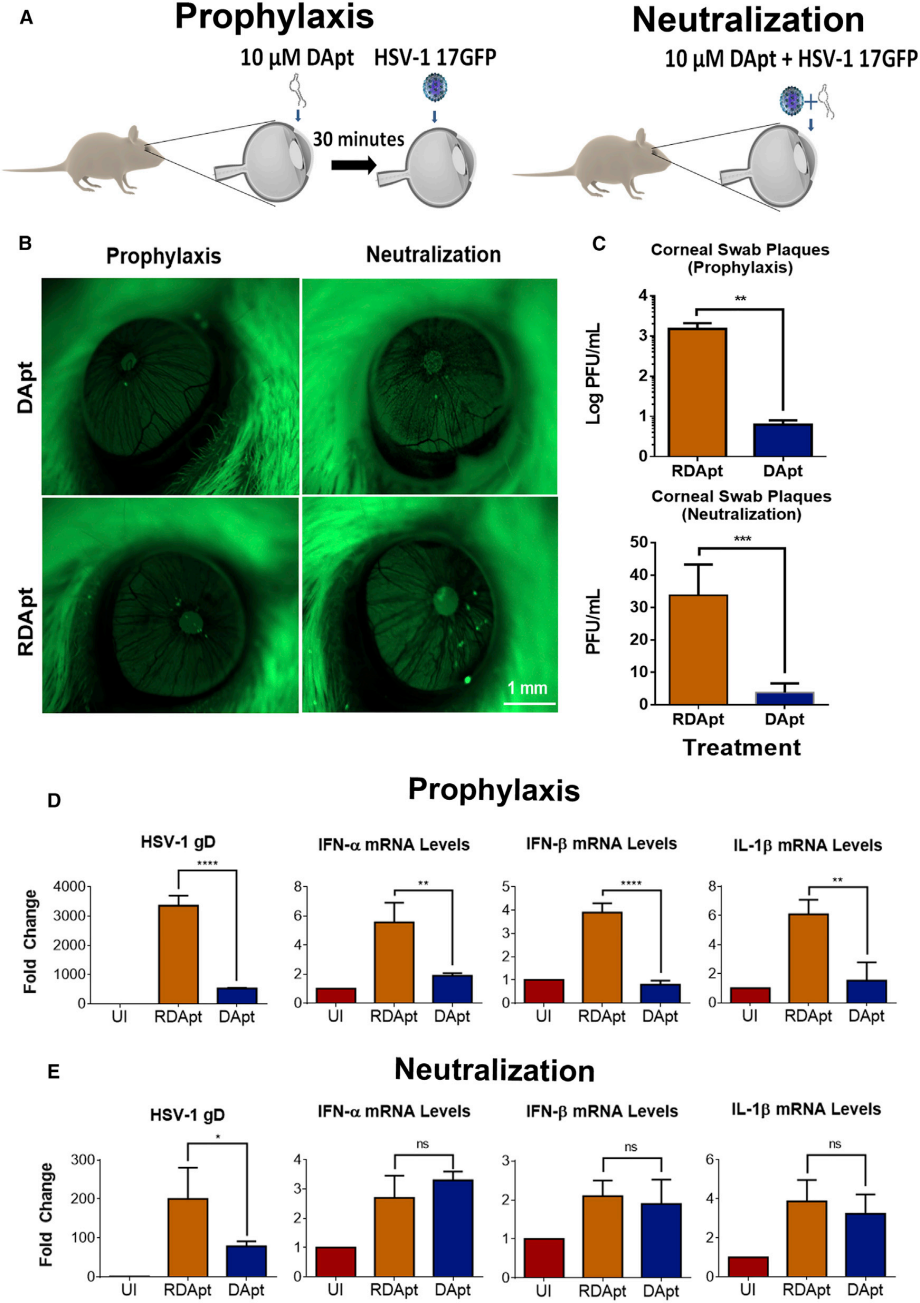
Prophylaxis and Neutralization Treatments of DApt Inhibit HSV-1 Infection in the *In Vivo* Model (A) A schematic of the prophylactic and neutralization treatments conducted on the mouse corneal models. Mice were sedated and the corneal epithelium was partially debrided using a 30G needle. For the prophylactic model, corneas were treated with 10 μM DApt/RDApt for 30 min before infecting with 10^6^ PFU HSV-1 (17-GFP). For the neutralization model, 10^6^ PFU HSV-1 (17-GFP) were incubated with 10 μM DApt/RDApt for 30 min and then added to the corneas. (B) Representative stereoscope images of the mouse cornea taken 48 hpi for the indicated treatments showing the presence of virus (green). Scale bars shown are for all the images. (C) Tears of the infected mice, in the form of corneal swabs, were collected 72 hpi, and a plaque assay was conducted with the same to understand the extent of infection in each mice. Plaque numbers for each treatment group are shown. Asterisks indicate significant difference by unpaired t tests: **p < 0.0021 (prophylaxis) and ***p < 0.0009 (neutralization). (D and E) Fold change in IFN-α, IFN-β, and IL-1β transcript levels in enucleated mouse eyes for prophylaxis (D) and neutralization (E) treatments quantified via qRT-PCR. Mouse corneal tissue was harvested 72 hpi. Asterisks indicate significant difference by one-way ANOVA with Dunnett’s multiple comparison test, with a single pooled variance: *p < 0.05, **p < 0.01, ***p < 0.001, and ****p < 0.0001. (C–E) Data are represented as means ± SD.

Based on the results we observed in the *ex vivo* models, we tested the prophylactic and neutralization ability of DApt to suppress HSV-1 infection in an intact animal (mouse) model (Figure 5A). Post epithelial debridement, mice eyes were treated with either DApt or RDApt according to prophylaxis or neutralization protocols prior to infection with HSV-1 17-GFP. We used a high virus titer (2 × 10^7^ plaque-forming units [PFU]) for these experiments to study the effect of DApt on viral entry at earlier time points. Representative stereoscope images taken at 48 hpi show that corneas treated with DApt had lower infection (green spots) than those treated with RDApt (Figure 5B). Tear samples collected from the mice eyes at 72 hpi were assayed to quantify the viral titers. In both prophylactic and neutralization treatments, we observed lower viral titers in the eye swabs of mice treated with DApt compared to RDApt (Figure 5C). Moreover, qPCR analysis of mRNA extracted from mouse corneal tissue showed significant reductions in the viral gD transcripts for prophylaxis (80% reduction) and neutralization (50% reduction) DApt treatments (Figures 5D and 5E). An interesting observation to note is the difference in infectivity between the prophylaxis and neutralization models. We observed more infection in the prophylaxis model compared to the neutralization model. This difference may be attributed to the experimental design. Although in the prophylaxis model, the eyes are first pre-treated with the treatments for 30 min and then infected, in the neutralization model, the virus and the treatments are mixed together for 30 min and then added to the eye. The process of mixing the aptamers with the virus might have resulted in non-specific binding between the negatively charged RDApt and virus, resulting in lower rates of infection compared to the prophylaxis model.

We also evaluated for cellular cytokine transcripts, such as IFN-α, IFN-β, and interleukin-1β (IL-1β), which are normally induced upon infection. qRT-PCR analysis revealed that DApt significantly reduced the induction of cytokine transcripts compared to RDApt only in the prophylaxis model, whereas no change was observed in the induction of the cytokine transcripts between the DApt- and RDApt-treated cells in the neutralization model, possibly because of the experimental design mentioned above (Figures 5D and 5E).

**Figure 6:**
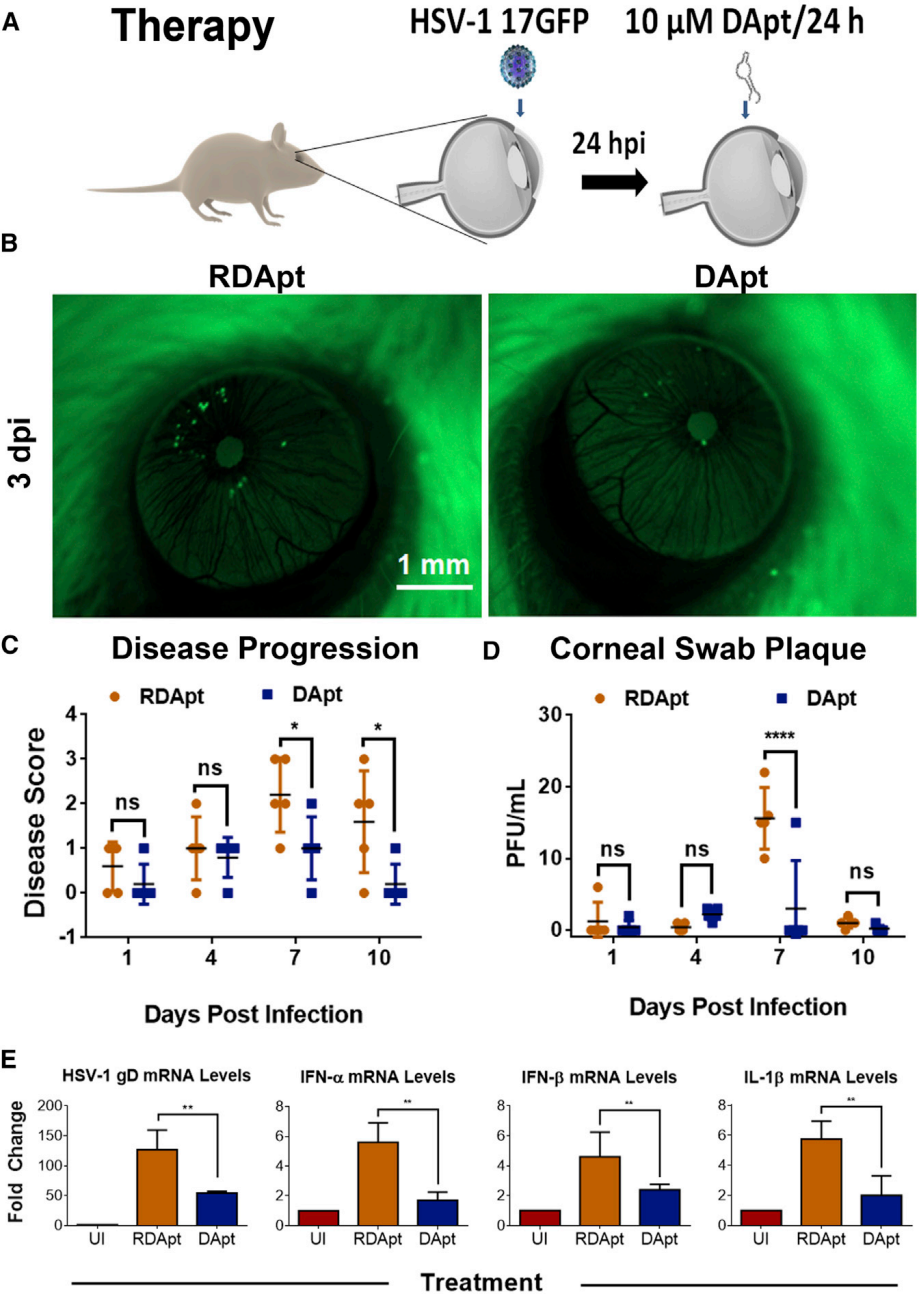
Therapeutic Treatment of DApt Inhibits HSV-1 Infection in the *In Vivo* Model (A) A schematic of the therapeutic treatment conducted on the mouse corneal models. Mice were sedated and the corneal epithelium was partially debrided using a 30G needle prior to infection with 10^7^ PFU of HSV-1(17-GFP). 24 hpi, the mice were given a dose of either 10 μM DApt or RDApt, followed by a dose every 24 hr until 72 hpi. (B) Representative stereoscope images taken at indicated times to show presence of virus (green). Scale bars shown are for all the images. (C) Animals were scored based on disease progression following HSV-1 (17-GFP) infection for a period of 14 days as follows: 0, no lesions; 1, minimal eyelid swelling; 2, moderate swelling; 3, moderate swelling with ocular discharge; 4, eyelid swelling with corneal opacity; and 5, severe swelling of the eyelid with hair loss and dendritic lesions. Asterisks indicate significant difference by two-way ANOVA with Sidak’s multiple comparison test: *p < 0.05. (D) Tears of the infected mice, in the form of corneal swabs, were collected at indicated times, and a plaque assay was conducted with the same to understand the extent of infection in each mice. Plaque numbers for each treatment group are shown. Asterisks indicate significant difference by two-way ANOVA with Sidak’s multiple comparison tests: ****p < 0.0001. (E) Fold change in IFN-α, IFN-β, and IL-1β transcript levels in enucleated mouse eyes quantified via qRT-PCR. Mouse corneal tissue was harvested 72 hpi. Asterisks indicate significant difference by one-way ANOVA with Dunnett’s multiple comparison test, with a single pooled variance: **p < 0.01 and ***p < 0.001. (C–E) Data are represented as means ± SD.

Because HSV-1 mostly spreads via cell to cell in corneal tissues and uses gD for this process, we wanted to test the therapeutic ability of DApt to specifically bind to gD and block HSV-1 infection. DApt or RDApt were topically applied to infected murine corneas at 24 hr post epithelial debridement and infection (Figure 6A). A 10-fold lower virus titer (2 × 10^6^ PFU) was used in the therapeutic model to study the effect of DApt treatment at later stages of viral infection (post entry) for a longer period of time (14 days). Representative stereoscope images taken 3 days post infection show that mice in both treatment groups were infected, albeit the DApt treatment group had a slightly lower amount of infection (Figure 6B). To further assess infection, tear samples collected from the mice eyes were assayed to titer the presence of virus. Interestingly, although no differences in virus titers were observed on day 4, a significant reduction of virus titers was observed on day 7 (Figure 6C). We are not sure why we do not see changes on day 4; however, the low viral titers seen during these experiments could be attributed to non-specific-charge-based neutralization, similar to those described above, by RDApt during our therapeutic treatment. We also monitored corneal disease progression by recording scores assessed by a blind observer. The DApt-treated mice consistently showed lower clinical scores compared to the RDApt-treated group and were significant at 7 and 10 dpi (Figure 6D). qPCR analysis of mRNA extracted from excised corneas at 14 dpi showed 45% lower viral gD transcripts with DApt treatment compared to RDApt treatment (Figure 6E). The IFN-α, IFN-β, and IL-1β response was also significantly lower with DApt treatment than with RDApt (Figure 6E). This could be attributed to the presence of lower infection in those treated with DApt as opposed to RDApt. These results correspond well with the cytokine levels recorded for the prophylaxis treatments (Figure 5D). Collectively, using a variety of treatment regimens *in vivo*, our findings suggest that DApt can effectively block HSV-1 viral infection and spread in the cornea, and together our findings provide promise for future use of DApt in clinical settings.

## Discussion

Only two aptamers have been superficially tested for efficacy against HSV in the past and both aptamers were RNA in composition.^13^, ^14^ Although RNA aptamers generally have a more versatile structure, enabling them to form multiple secondary structures,^25^ their stability in biological systems is low and further compounded by a high degradation rate at room temperature or higher. Multiple stabilization methods have been proposed for RNA;^26^ however, they have shown to weaken their ability to bind to desired targets and also to negatively impact cost effectiveness. DNA is a comparatively stable molecule, with respect to biological systems,^27^ although its versatility is not considered equivalent to RNA. In this work, a DApt was designed based on the mini-1 RNA aptamer, which is already shown to bind HSV-1 gD with very strong affinity by Gopinath et al.^13^ To our surprise, the shorter DNA preserves key structural features of its native RNA form and, most significantly, it binds gD, albeit with lower affinity than the parent RNA aptamer. Our serendipity with DApt was observed when we used the DNA clone as a control for testing the antiviral activity of the parent RNA aptamer (Figure S2C). To our surprise, the DNA form had good stability and high anti-HSV-1 activity in *ex vivo* models. Furthermore, the cost of the DNA form was 10-fold cheaper than its RNA parent. Although preliminary experiments (Figure S2C) with the parent RNA aptamer were conducted, this study was geared toward understanding the efficacy of DApt in controlling HSV-1 infection. Overall, our experiments suggest that DApt can provide a suitable alternative to many conventional designs and provide an effective strategy for viral glycoprotein-targeting drug discovery efforts.

The results obtained from the viral entry assay show 50%–80% reduction in viral entry through neutralization of the virus by DApt, with an EC_50_ of 2 μM, as opposed to RDApt (Figure 2). The 24-hr viral replication study showed similar results, reiterating the role of DApt in reducing initial viral entry corresponding to lower infectious spread (Figures 3A–3C). Furthermore, the ability of DApt to inhibit cell fusion signifies that this aptamer has a multifaceted role in not only reducing initial viral entry but also restricting intracellular viral spread via blocking syncytia formation (Figures 3D–3F).The DApt did not cause any toxic effects and did not induce any unusual cytokine responses, suggesting that the antiviral activity of DApt was through specifically binding to gD (Figures 1D and 1E). HSV-1 gD protein interacts with host membrane receptors, HVEM and Nectin-1, to facilitate entry into cells. Although the affinity of interactions between gD and HVEM/Nectin-1 is in the low micromolar range, it has been shown that the binding affinity of an interfering molecule to effectively disrupt HVEM/Nectin-1-gD interactions needs to be in the nanomolar range (40 nM).^13^ Our *in silico* results have shown that the binding affinity of DApt is also in the comparable range (50 nM), which is strong enough to competitively interfere with gD/receptor interactions. It is also important to note that although the IDT Oligo-analyzer tool suggests similarities in structural characteristics between DApt and its parent mini-1 RNA aptamer, many other differences, including inhibitory effects due to charged nature of the oligomers causing a “heparin” like effect, may influence the virus inhibitory properties of the aptamer. All of this will be thoroughly analyzed in our future studies that will also map out the binding sites of the aptamer on gD crystal structure.

An interesting aspect of using aptamers in therapy is that aptamer-binding affinities change with variations in salt content, pH, and temperature.^28^, ^29^ Although examining this possibility with our treatment, we noted that during *in vitro* experiments, when DApt was compared to acyclovir or TFT (trifluorothymidine), the therapeutic effects of the aptamer were not as strong, especially when the treatments were started 2 hpi for a period of 24 hr (Figure S3A). To understand this anomaly further, we performed neutralization experiments using aptamers dissolved in different buffers. As suspected, DApt had no virus neutralizing ability when the salt concentration in buffers was changed, which may be a limitation of our approach. However, as discussed below, to our satisfaction, our original aptamer formulation in PBS showed excellent results in *ex vivo* cornea cultures and murine ocular infections. When DApt was tested for its efficacy in restricting viral entry in porcine corneal tissue cultures infected with GFP-tagged HSV-1 (17-GFP), it was clear that viruses neutralized with DApt showed a lower radial spread compared to mock or RDApt treatments (Figure 5).

Similar therapeutic effects were seen when the aptamer was applied as a topical eye drop over porcine corneal tissues post HSV-1 infection (Figure 4). Noticeably, the infectious spread of the GFP virus was contained throughout the treatment period and regained when the treatment was stopped. This could be because DApt is able to restrict newly produced viral particles from entering nearby uninfected cells while also restricting cell-to-cell fusion and thereby inhibiting intracellular viral spread. Also, the pH and salt concentrations of the excised cornea might actually be complementing DApt’s neutralization ability, which were lacking in the *in vitro* culture model. Mock- and RDApt-treated samples were observed to have dendritic lesions that continue to form over a period of 10 days. However, the limitation of the aptamer treatment was that it was not able to completely eliminate the presence of the virus from the corneal tissue.

To further test the efficacy of DApt in *in vivo* models, mouse corneal tissue was infected with GFP virus pre-treated either prophylactically or through neutralization (Figure 5). The studies showed the protective ability of DApt in restricting viral entry and spread in corneal tissue when applied prophylactically. Mice pre-infected and then treated with DApt showed lower disease scores, viral titers, and viral RNA transcripts, suggesting its role in reducing HSV-1 infection *in vivo*. However the results obtained in the *in vivo* therapeutic model were not as prominent as the *ex vivo* therapeutic model or *in vivo* prophylactic model we conducted. This could be attributed to lower retention time or change in aptamer concentration on the corneal surface *in vivo* compared to *ex vivo* corneas and the extent of viral spread to the deeper layers of the cornea during *in vivo* infection. We believe that although our DApt is an excellent entry inhibitor that shows potential prophylactic therapy against HSV-1 infection, further improvements involving extended retention/release models would make it an attractive candidate as a therapeutic against ocular herpes infection.

In conclusion, this is the first study to show a comprehensive decrease in HSV-1 infection using a DNA aptamer. The 45-nt DApt was found to bind to HSV-1 surface glycoprotein gD and was able to restrict viral entry into host cells. Its role in inhibiting cell-to-cell fusion and consequently restricting viral spread was also established in this study. Furthermore, its role as a therapeutic agent was investigated using both *ex vivo* and *in vivo* models. Although DApt shows significant therapeutic efficacy *in vivo*, our results suggest that as an entry inhibitor, it would be able to show greater efficacy when used synergistically with other topical therapeutics, such as TFT, ganciclovir, or inhibitors of heparanase, which can reduce viral release and resultant pathogenesis.^30^, ^31^ These studies will be part of our future work, which will also include studies directed toward determining the structural differences between the DNA and RNA aptamers and mapping out the aptamer-binding sites on gD. Future studies will also shed more light on the clinical applicability of our aptamer.

## Materials and Methods

### Cells, Virus, Media, and Plasmids

Human corneal epithelial cells (RCB1834 HCE-T) were obtained from Kozaburo Hayashi (National Eye Institute, Bethesda, MD) and were cultured in minimum essential medium (MEM) (Life Technologies, Carlsbad, CA) with 10% fetal bovine serum (FBS) (Sigma-Aldrich, St. Louis, MO) and 1% penicillin/streptomycin (P/S) (Life Technologies). The African green monkey kidney (VERO) cell lines were obtained from Dr. Patricia G. Spear (Northwestern University, Chicago, IL) and cultured in DMEM (Life Technologies) with 10% FBS and 1% P/S. CHO-K1 cells were provided by P. G. Spear (Northwestern University). CHO-K1 cells were passaged in Ham’s F12 medium (Gibco/BRL, Carlsbad, CA, USA) supplemented with 10% FBS and P/S (Sigma).

All the oligo sequences, including DApt, RDApt, RNA aptamer, and FAM-tagged DApt, were purchased from IDT. Aptamers were used as received and dissolved in PBS (DApt/RDApt) or Tris-HCl (RNA Aptamer) and stored at −20°C. Aliquots of 100-μL aptamers were heated to 95°C for 3 minutes and cooled on ice prior to use in any experiments. Trifluorothymidine (TFT) and acyclovir (ACV) were purchased from Selleckchem, and stock solutions were prepared in DMSO and stored at −20°C.

Three strains of HSV-1 were used: wild-type HSV-1 KOS; β-galactosidase-expressing HSV-1 reporter virus (gL86); and GFP-tagged HSV-1 17-GFP (purified using sucrose gradient). MEM (Gibco) and OptiMEM (Gibco) were used in the 6-hr and 24-hr infection models. DMEM (Gibco) mixed with 5% methyl cellulose (Sigma-Aldrich) was used for obtaining plaque assays. Plasmids for gB, gD, gH, gL, Luciferase gene, T7 promoter sequence plasmids (synthesized by standardized protocols [Promega]), and F12 media (Gibco) were used in a cell-to-cell fusion assay. Soluble gB, gD, and nectin-1 proteins were kindly provided by G. H. Cohan (University of Pennsylvania, PA) and R. J. Eisenberg (University of Pennsylvania, PA).

### SYBR Green Assay

This assay is based on the ability of SYBR Green (SG, N’,N’-dimethyl-N-[4-[(E)-(3-methyl-1,3-benzothiazol-2-ylidene)methyl]-1-phenylquinolin-1-ium-2-yl]-N-propylpropane-1,3-diamine) to fluoresce in the presence of a double-stranded DNA molecule as an intercalation dye.^19^ Briefly, 50 μL of multiple concentrations (200 nM to 1 nM) of soluble gD, gB, and nectin-1 protein were dispensed into a 96-well plate. DApt (4 μL) was added to each well, and the samples were incubated for a period of 30 min before 4 μL SG was added to each well. DApt with SG, DApt alone, and SG alone were used as controls for the reaction. The fluorescence recorded at 520 nm was used to the calculate the binding affinity using Equation 1,

(Equation 1) $$Affinity=\;\frac{f0-f1}{f0},$$

where f_0_ is the fluorescence intensity of SG and DApt in the absence of any protein, whereas f1 is the fluorescence emitted by SG and DApt in the presence of protein. Because this equation would give us the total amount of aptamer bound to the protein, a non-linear regression analysis of the determined values was generated to calculate the K_d_ for DApt. GraphPad Prism software was used to generate the K_d_ values using triplicates of the experiment.

### FAM-Tagged DApt Assay

CHO cells (plated on glass-bottomed dishes) were transfected with either a control plasmid (empty vector), Nectin-1 plasmid, gB plasmid, or gD plasmid (1.0 μg/mL) using standard lipofectamine protocols and incubated for a period of 24 hr at 37°C at 5% CO_2_. The cells were then permeabilized with 4% paraformaldehyde (Electron Microscopy Sciences, PA, USA) for 30 min and stained with DAPI (NucBlue, Molecular Probes, USA) for 10 min. Preheated and cooled 2 μM FAM-tagged-DApt (purchased from IDT) was then added to the CHO cells and incubated for a period of 30 min before they were washed twice with PBS. The cells were imaged at 63x on a laser confocal microscope (Leica, SP2) using z stack full image projection.

### MTT Cytotoxicity Assay

DApt and RDApt oligonucleotides were tested for their toxicity by evaluating cellular mitochondrial activity 24 hr post exposure. Briefly, HCEs were plated in a 96-well plate at a seeding density of 2 × 10^4^ per well and left overnight until they were 80% confluent. Various concentrations of pre-heated (and cooled) DApt, RDApt, and PBS were added to each well and allowed to incubate for a period of 24 hr at 37°C and 5% CO_2_. Post incubation, wells were washed with PBS twice before 100 μL 0.5 mg/mL MTT was added to each well and incubated for 4 hr. Formazan crystals that formed due to the mitochondrial activity were dissolved using acidified isopropanol (0.1% HCl in isopropanol) and transferred to a new 96-well plate. The color developed was analyzed by a Tecan GENios Pro microplate reader at 562 nm. Experiments were conducted in triplicates and individually repeated 5 times.

### qPCR

This protocol was used to extract cellular RNA and quantify cellular transcript levels, specifically, GAPDH, IFN-α, IFN-β, and IL-1β. Viral gD RNA transcripts were also quantified using this method in order to evaluate total infection in HCEs and mouse corneal tissues. The process is similar to those described in our previous reports,^32^ in which RNA was extracted from cells using TRIzol (Life Technologies) according to the manufacturer’s protocol. RNA was then transcribed to cDNA using the High Capacity cDNA Reverse Transcription Kit (Applied Biosystems, Foster City, CA). Real-time qPCR was performed with Fast SYBR Green Master Mix (Applied Biosystems) using QuantStudio 7 Flex (Applied Biosystems). The primers used in this study are listed in Tables 1 and 2.

**Table 1:** List of Primers Used for Amplifying cDNA Transcripts of RNA Extracted from HCEs

Target	Direction	Sequence (5′–3′)
GAPDH	forward	CAC CAC CAA CTG CTT AGC AC
	reverse	CCC TGT TGC TGT AGC CAA AT
IFN-α	forward	GAT GGC AAC CAG TTC CAG AAG
	reverse	AAA GAG GTT GAA GAT CTG CTG GAT
IFN-β	forward	CTC CAC TAC AGC TCT TTC CAT
	reverse	GTC AAA GTT CAT CCT GTC CTT
TNF-α	forward	AGC CCA TGT TGT AGC AAA CCC
	reverse	GGA CCT GGG AGT AGA TGA GGT
IL-1β	forward	TCG CCA GTG AAA TGA TGG CT
	reverse	TGG AAG GAG CAC TTC ATC TGT T
gD	forward	TAC AAC CTG ACC ATC GCT TC
	reverse	GCC CCC AGA GAC TTG TTG TA

**Table 2:** List of Primers Used for Amplifying cDNA Transcripts of RNA Extracted from Mouse Tissue

Target	Direction	Sequence (5′–3′)
GAPDH	forward	CCT GCT GGC TGT GAG GAA AT
	reverse	GAC AGG GCT CTC CAG ACT TC
IFN-α	forward	CCT GCT GGC TGT GAG GAA AT
	reverse	GAC AGG GCT CTC CAG ACT TC
IFN-β	forward	TGT CCT CAA CTG CTC TCC AC
	reverse	CAT CCA GGC GTA GCT GTT GT
IL-1β	forward	GTG GCT GTG GAG AAG CTG TG
	reverse	GAA GGT CCA CGG GAA AGA CAC

### Virus Neutralization by Aptamers

Neutralization experiments were performed by adding the required amount of virus and pre-heated and cooled DApt or RDApt in 400 μL OptiMEM, followed by incubation for 30 min, with constant agitation at room temperature. At the end of 30 min, the mixture of the virus and DApt were added to cells. 30-min neutralization allows for attaining adsorption equilibrium between the aptamers and virus glycoproteins. PBS was added to the virus solution and incubated for 30 min in the mock-treated samples.

### Viral Entry Assay

The viral entry assay was performed using protocols previously established.^22^ HCEs were plated at a seeding density of 2 × 10^4^ per well in a 96-well plate and were left overnight until they were 90% confluent. 0.1 μL/well (MOI 10) of gL86 virus (2 × 10^8^ PFU/mL stock) solution was neutralized by PBS, RNA aptamer, DApt, or RDApt before they were added onto the monolayer of cells. 6 hr post infection, the wells were cleaned twice with PBS before the 100 μL β-galactosidase substrate (0.5% Nonidet P40 and 3 mg/mL ONPG, o-nitro-phenyl-β-d-galactopyranoside; ImmunoPure, PIERCE, Rockford, IL) solution was added to each well. The plates were stored at 37°C for a period of 2 hr before the enzymatic activity was analyzed using a GENESIS Pro Plate reader at 410 nm.

Viral entry assay was also evaluated through immunoblotting for HSV-1 ICP-0, an early gene product made immediately upon viral entry. HCEs were plated at a seeding density of 1.2 × 10^6^ per well in a 6-well plate and used when the cells reached 80% confluency. HSV-1 (KOS) at an MOI of 10 was neutralized by either PBS/DApt/RDApt for a period of 30 min at the EC_50_ (2 μM) determined by the β-galactosidase assay mentioned above. Cells were infected for a period of 2 hr before fresh media was added to the cells. At 6 hpi, cells were collected, lysed, and immunoblotted for HSV-1 ICP0 protein and quantified using ImageJ software.

### Viral Replication Assay

HCEs were plated at a seeding density of 1.2 × 10^6^ per well in a 6-well plate. Neutralization was performed by incubating ACV/DApt/RDApt/mock (at indicated concentrations) with HSV-1 KOS (or TK-12) virus (0.6 μL of 2 × 10^8^ PFU/mL stock) for a period of 30 min before they were added to the cell monolayer. At 2 hpi, cells were washed with PBS, replenished with MEM, and incubated overnight for 24 hr before the cells were lysed and immunoblotted for HSV-1 gD.

### Flow Cytometry

HCEs were plated at a seeding density of 2 × 10^5^ per well in a 24-well plate. HSV-1 17-GFP virus (0.1 MOI) neutralization was performed by incubating with either DApt or RDApt for a period of 30 min at indicated concentrations (0–10 μM). Post neutralization, virus/aptamer solution was added to the cell monolayer and incubated at 37°C at 5% CO_2_ for 2 hr. Subsequently, cells were washed with PBS twice and fresh MEM media was added to the cells. At 24 hpi, cells were washed carefully with PBS and imaged with a stereoscope under the GFP channel prior to preparing the cells for flow cytometry. Cells were then dislodged by adding 100 μL trypsin to each well for a period of 10 min and washed with PBS twice through centrifugation at 4,000 rpm at 4°C. The cell pellet was washed once in FACS buffer (PBS with 2% FBS) before it was suspended in 300 μL FACS buffer. Cells were analyzed using a BD Accuri C6 plus instrument under the live/singlet gates, with 25,000 events per sample. All experiments were done in quadruplicates.

### Immunofluorescence Imaging

In order to visualize the virus-restricting capabilities of DApt, HCEs were plated at a low seeding density (1.2 × 10^5^ cells/well) in glass bottom imaging dishes (MatTek, Ashland, MA, USA). Neutralization treatment using 10 μM DApt was performed at a high MOI (10), and the virus solution was added to the cells and incubated at 4°C for a period of 2 hr to allow viral adsorption. Entry was initiated by incubating the plate at 37°C for 30 min. The cells were fixed in 4% paraformaldehyde (Electron Microscopy Sciences, Hatfield, PA, USA), permeabilized with 0.01% Triton-X (Thermo Fisher Scientific), and stained with 4′,6-diamidino-2-phenylindole (DAPI; Life Technologies) to stain the nucleus. The images were captured under 63x objectives using an observer microscope (Carl Zeiss, Jena, Germany) with a spinning disk (CSU-X1; Yokogawa, Tokyo, Japan).

### Immunoblotting

Virally infected cell lysates were denatured in NuPAGE LDS Sample Buffer (Invitrogen, NP00007) and heated to 80°C for 10 min. Equal amounts of protein were added to 4%–12% SDS-PAGE gel and transferred to a nitrocellulose membrane. Nitrocellulose membrane was blocked in 5% nonfat milk in Tris-buffered saline (TBS) for 2 hr at room temperature. After the nonspecific binding blocking step was complete, membranes were incubated with primary antibodies (mouse anti-ICP0 for the 6-hr infection model; mouse anti-gD monoclonal antibody [Abcam] for the 24-hr infection model) at dilutions of 1:1,000 overnight at 4°C. The following day, the blots were washed multiple times with 0.1% TTBS (0.1% Tween 20 in TBS) before the addition of horseradish peroxidase conjugated anti-mouse IgG at dilutions of 1:25,000 at room temperature. Protein bands were visualized on an ImageQuant LAS 4000 imager (GE Healthcare) after the addition of SuperSignal West Pico maximum sensitivity substrate (Pierce, 34080). The density of the bands was quantified using ImageQuant TL image analysis software (version: 7). GAPDH was measured as a loading control.

### Cell-to-Cell Fusion Assay

A standard virus-free cell-to-cell fusion assay was performed as described previously.^21^ Two populations of CHO-K1 cells, designated target cells and effector cells, were generated. Although the target cell population was transfected with nectin-1 (1.0 μg) and plasmid expressing the luciferase gene (0.5 μg), the effector cell population was transfected with HSV-1 glycoproteins gB, gD, gH, and gL and T7 RNA polymerase (0.5 μg each in 6-well plates). Effector cells without gB plasmid were used as a negative control because they would not contribute to active cell-to-cell fusion. After transfection, effector and target cells were mixed in a 1:1 ratio and co-cultured in 24-well plates. PBS, DApt, or RDApt at a final concentration of 2 μM was added to these mixtures. Luciferase gene expression resulting from fusion of target and effector cells, 24 hr post mixing, was measured using a reporter lysis assay (Promega). All the experiments were performed in triplicates, and the plates were imaged to observe syncytia formation using a live cell nucleus stain (Molecular Probes NucBlu; R37605) at 10x magnification.

### *Ex Vivo* Porcine Corneal Infection Model

Freshly sacrificed pig eyes were collected from a local butcher shop and used no later than 24 hr. Two needle pokes were presented on each cornea using a 25-mm 30G needle (BD Precisionglide) before the cornea was carved from the eye using a surgical blade. The corneas were cleaned multiple times in PBS mixed with 5% antifungal antibacterial (Gibco) solution before they were placed in a 12-well plate.

Neutralization treatment was performed by incubating DApt, RDApt, or mock (PBS) (final concentration, 10 μM) with 5 × 10^6^ PFU HSV-1 17-GFP virus. 30 min post incubation, the solution was added to each cornea and left to infect for a period of 24 hr in cornea media (MEM with 5% antifungal antibacterial and 1% insulin transferrin [Sigma]). 24 hr later, the corneas were washed with PBS twice and the media was replenished. Corneas were washed and imaged every 24 hr for a period of 3 days. Quantification of the porcine corneal infection in the case of neutralization studies was done using ImageJ software. The brightness and contrast of the images were maintained constant, and the threshold option was used to select only the infectious zones. Once selected, the area of the selected zones was combined and calculated to represent pixel counts.

Therapeutic treatment was initiated 48 hr post infection of the corneas with 5 × 10^6^ PFU HSV-1 17-GFP virus. All the corneas were washed with PBS and imaged using Zeiss SteREO Discovery.V20 at a constant exposure time of 400 ms at a magnification of 7.5× for the presence of GFP virus. Triplicates of corneas were either treated with PBS, DApt, or RDApt by adding 100 μL 10 μM DApt onto the corneal poke site, followed by addition of cornea media (400 μL). The treatment was repeated every 24 hr for a period of 10 days, and images of the cornea were collected subsequently before the addition of treatment solutions.

To understand if stopping DApt treatment would increase viral infection, 7 days post initial infection, DApt treatment was stopped in one set of corneas, whereas it was continued in another set. The spread of virus was monitored by imaging both sets of corneas every 24 hr for period of 3 days. At the end of 10 days, the corneas were discarded after the addition of 10% bleach solution to each cornea.

### Mouse Cornea Infection

6- to 8-week-old male and female BALB/c mice obtained from Charles River Laboratories (Wilmington, MA) were housed at the University of Illinois at Chicago Animal Facility and used for all animal experiments. Mice were anesthetized using ketamine (100 mg/kg) and xylazine (5 mg/kg) prior to the application of proparacaine hydrochloride ophthalmic solution (Alcon Laboratories, TX, USA), and epithelial debridement of the right eye with a 30G sterile needle in a 3 × 3 grid pattern, as previously reported.^33^ The level of anesthesia was determined by loss of toe pinch/pedal withdrawal. Animals were maintained under a heat lamp until they recovered from anesthesia. 1 μL 10 μM DApt/RDApt/PBS was either used to neutralize HSV-1 17-GFP virus (2 × 10^7^ PFU) or applied directly onto the cornea (prophylaxis) for 30 min. The virus solution was then added onto the cornea to initiate infection. Mice were monitored every 24 hr and imaged every 48 hr for a period of 10 days to record any changes occurring due to infection. Tear samples were collected using a calcium alginate tipped Calgiswab dipped in 1 mL DMEM media, which were swabbed on and around the eye 3 times to collect replicating virus from the cornea. Animals were observed daily for complications and their weights were monitored closely. Mice demonstrating pain or suffering were euthanized. Before euthanasia, mice were injected intraperitoneally with a cocktail of ketamine (100 mg/kg) and xylazine (5 mg/kg) via intraperitoneal injection and were cervically dislocated. This method is consistent with the recommendations of the Panel on Euthanasia of the American Veterinary Medical Association. Ten mice (5 male; 5 female) per treatment group were used for the experiment.

To evaluate the therapeutic efficacy of DApt in the *in vivo* animal model, mice were anesthetized as described above and their right eyes were subjected to epithelial debridement prior to the application of HSV-1 17-GFP virus (2 × 10^6^ PFU). The mice were left untreated for a period of 24 hr before 5 μL 10 μM DApt/RDApt/PBS was added to their eyes. Mice were imaged every 24 hr until 72 hr using Zeiss SteREO Discovery.V20 at a constant exposure time of 400 ms to check for the presence of GFP virus. Ten mice (5 male; 5 female) per treatment group were used for the experiment.

### Statistical Methods

All the statistical analysis conducted in the manuscript was performed using GraphPad Prism Software Version-6. All the error bars represent mean ± SD, which were automatically calculated by the software. All one-way ANOVA analysis used Dunnett’s multiple comparison tests with a single pooled variance. All two-way ANOVA analysis used Sidak’s multiple comparison tests. Plaque numbers from the *in vivo* corneal swab were analyzed using unpaired t tests.

### Ethics Statement

Animal care and procedures were performed in accordance with institutional and NIH guidelines, and approved by the Animal Care Committee at the University of Illinois at Chicago. The Biologic Resources Laboratory of the University of Illinois at Chicago has a modern animal facility, with several veterinarians on staff available for expert veterinary care and advice during the project. The Animal Care Committee (ACC) at the University of Illinois at Chicago approved the animal experiments under the permit no. ACC15-091.

## Author Contributions

T.Y., A.A., D.J., K.M., and N.T. conducted the experiments; T.Y., K.P., and D.S. designed the experiments. T.Y., D.J., A.A., and D.S. wrote the paper.

## Supplemental Information

Document S1. Figures S1–S3

Document S2. Article plus Supplemental Information
