# Supplementary material for: Targeting Herpes Simplex Virus-1 gD by a DNA Aptamer Can Be an Effective New Strategy to Curb Viral Infection
Source: Mol Ther Nucleic Acids. 2017 Oct 17;9:365–78. doi: 10.1016/j.omtn.2017.10.009 (PMC5686428; doi:10.1016/j.omtn.2017.10.009)
Supplement: Document S1. Figures S1–S3 [file mmc1.pdf]

OMTN, Volume *9*

Supplemental Information

Targeting Herpes Simplex Virus-1 gD by a DNA Aptamer Can Be an Effective New Strategy to Curb Viral Infection

Tejabhiram Yadavalli, Alex Agelidis, Dinesh Jaishankar, Kyle Mangano, Neel Thakkar, Kumar Penmetcha, and Deepak Shukla

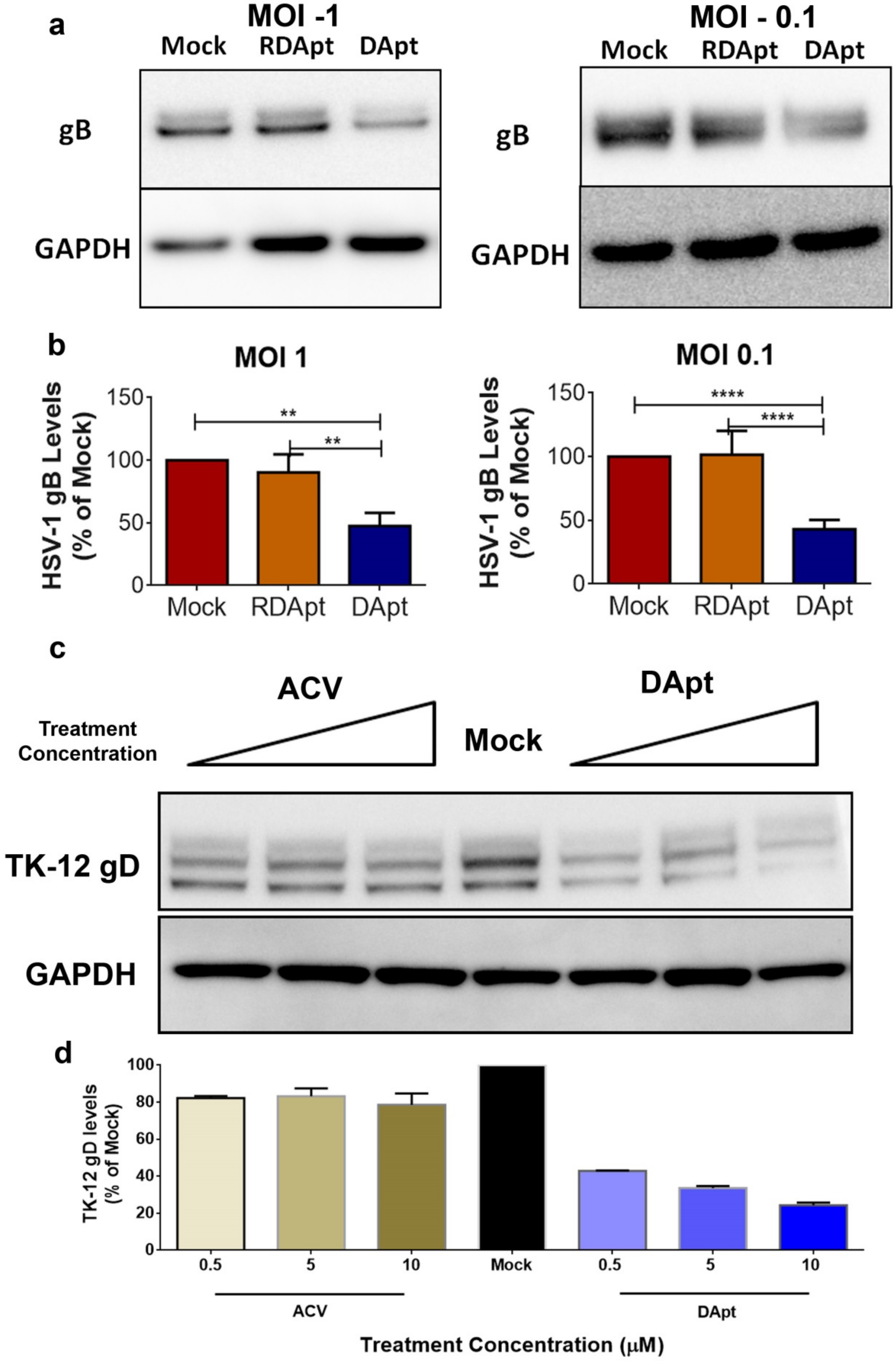
a
MOI -1
Mock RDApt DApt
gB
GAPDH
MOI - 0.1
Mock RDApt DApt
gB
GAPDH
b
MOI 1
HSV-1 gB Levels (% of Mock)
150
100
50
0
**
**
Mock RDApt DApt
MOI 0.1
HSV-1 gB Levels (% of Mock)
150
100
50
0
****
****
Mock RDApt DApt
c
ACV
Treatment Concentration
Mock
DApt
TK-12 gD
GAPDH
d
TK-12 gD levels (% of Mock)
100
80
60
40
20
0
0.5 5 10 Mock 0.5 5 10
ACV
DApt
Treatment Concentration (μM)

Supplementary figure S1: DApt neutralizes acyclovir resistant virus. (a) HSV-1 (KOS) at MOI 1 and 0.1 were neutralized with 2 µM DApt/RDApt for a period of 30 minutes before they were allowed to infect HCEs. At 2 hpi, cell monolayer was washed with PBS twice and fresh MEM media was added. 24 hpi, cells were collected, lysed and immunoblotted for HSV-1 gB to evaluate effective infectious levels. **(b)** The immunoblots were analyzed and quantified using image J software. **(c)** HSV-1 (TK-12) virus which is HSV-1 Thymidine Kinase null (molecular target for acyclovir) was neutralized with Acyclovir/Mock/DAPt at indicated concentrations for a period of 30 minutes before they were allowed to infect monolayer of HCE cells. At 2 hpi, cells were washed with PBS twice and fresh MEM media was added. 24 hpi, cells were lysed and immunoblotted for the presence of HSV-1 gD. **(d)** Immunoblots were quantified and analyzed using Image J software and plotted using GraphPad Prism software.

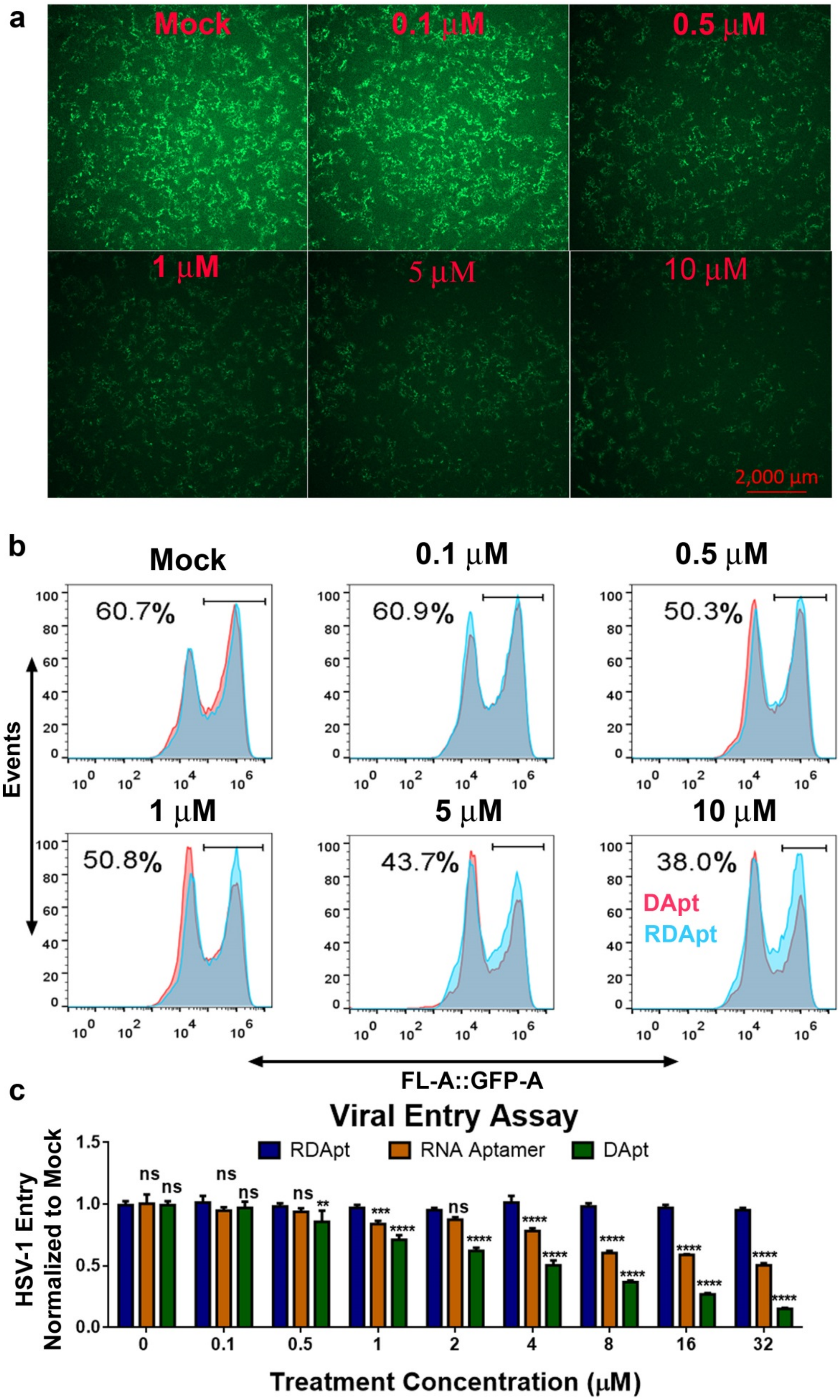
a
Mock
0.1 μM
0.5 μM
1 μM
5 μM
10 μM
2,000 μm
b
Mock
0.1 μM
0.5 μM
1 μM
5 μM
10 μM
60.7%
60.9%
50.3%
50.8%
43.7%
38.0%
DApt
RDApt
Events
FL-A::GFP-A
c
Viral Entry Assay
RDApt
RNA Aptamer
DApt
HSV-1 Entry
Normalized to Mock
Treatment Concentration (μM)
0
0.1
0.5
1
2
4
8
16
32
ns
**
***
****

Supplementary Figure S2. DApt neutralizes HSV-1 virus in-vitro. (a) HSV-1 (17-GFP) was neutralized with increasing concentrations of DApt/RDApt for a period of 30 minutes before they were allowed to infect HCE cells for 2 hours in a 24 well plate. At 2 hpi, cells were washed with PBS twice and fresh MEM media was added. At 24 hpi, cells were washed with PBS and imaged using Zeiss Stereoscope (7x magnification; 400 ms exposure) in the GFP channel. **(b)** Cells were then trypsinized, collected and washed with FACS buffer (2% fetal bovine serum in PBS) and filtered through a 63 micron nylon filter to remove any aggregates from the sample. 300 µL samples were analyzed using a BD Accuri C6 Plus Flow cytometer at 25,000 gated events for singlet cells. The data was analyzed using FloJo software. **(c)** Viral Entry assay was performed using HSV-1 gL 86 β-galactosidase producing reported virus, neutralized either by pre-heated (90 °C) and cooled RNA Aptamer (dissolved in 50 mM Tris-HCl, 50 mM KCl [pH 7.5]), DApt or RDApt (dissolved PBS) at indicated concentrations for 30 minutes. Neutralized virus was added to HCEs plated in a 96 well plate and incubated for 6 hours before the cells were lysed and suitable substrate (0.5% Nonidet P40 and 3 mg/mL ONPG, o-nitro-phenyl- β-d-galactopyranoside; ImmunoPure, PIERCE, Rockford, IL) solution was added to each well. The plates were stored at 37 °C for a period of 2 hours before the enzymatic activity was analyzed using a GENESIS Pro Plate reader at 410 nm. *Asterisks* indicate significant difference by two-way ANOVA with Sidak's multiple comparison test: $^{**}p<0.01$, $^{***}p<0.001$ and $^{****}p<0.0001$

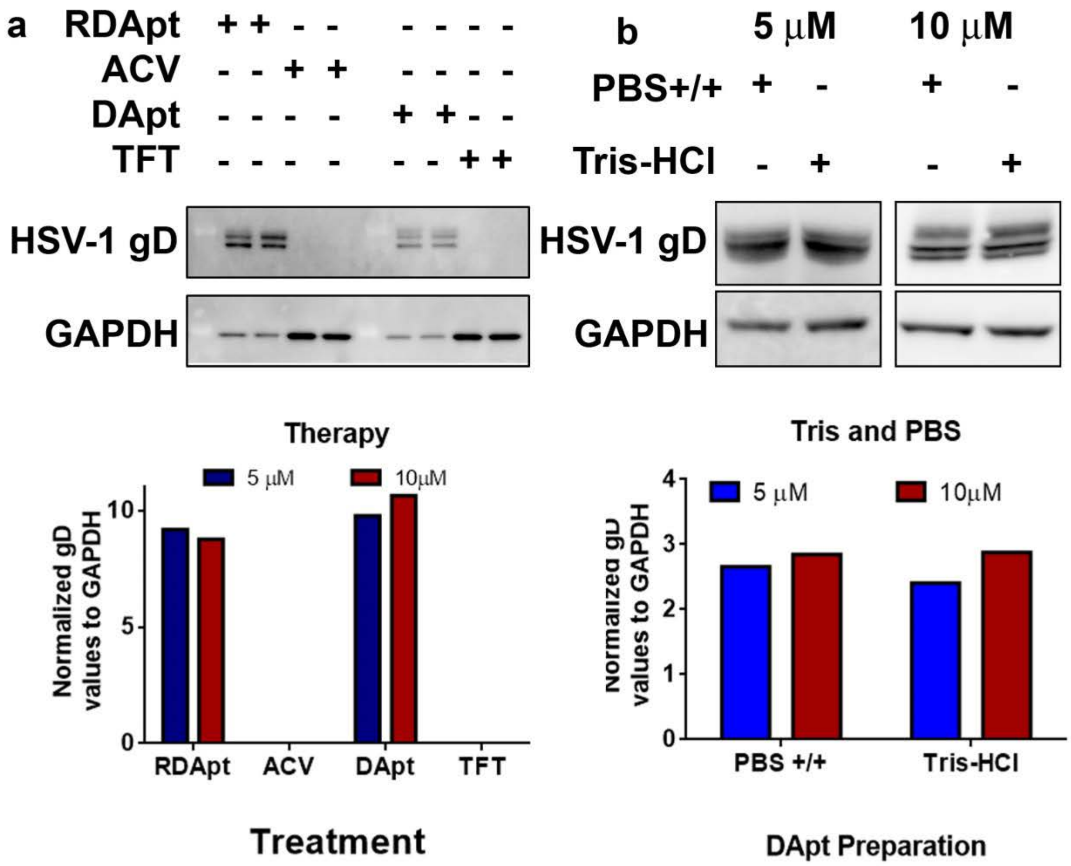

Supplementary Figure S3. DApt shows minimal therapeutic efficacy *in vitro*. (a) HSV-1 (KOS) at MOI 1 was used to infect HCE cells. At 2 hpi, cells were washed with PBS twice and MEM media with indicated concentrations of either RDApt/ACV/DApt/TfT was added to the cell monolayer. The treatments were incubated with the cells overnight and at 24 hpi, cells were lysed and immunoblotted for the presence of HSV-1 gD protein. The blots were analyzed using Image J software and the quantifications are shown. **(b)** To understand the difference in neutralizing ability of the DApt while dissolved in different buffers, indicated concentrations of DApt/RDApt were dissolved in Tris buffer (50 mM Tris-HCl, 50 mM KCl [pH 7.5]) or PBS +/+ (gibco, Life Technologies; $CaCl_2$ and $MgCl_2$ [pH 7.0]). Pre-heated and cooled aptamers were used to neutralize HSV-1 (KOS) at MOI 1 for 30 minutes before infecting HCE cells. At 2 hpi, cells were washed with PBS twice and fresh MEM media was added. At 24 hpi, cells were lysed and immunoblotted for HSV-1 gD. The blots were analyzed using Image J software and the quantifications are shown.
